# Association between stress hyperglycemia ratio and ICU delirium among critically ill adults in MIMIC-IV

**DOI:** 10.1038/s41598-026-40380-2

**Published:** 2026-02-17

**Authors:** Chong Wang, Lili Lv, Rongrong Ma, Haiyan Dong

**Affiliations:** Department of Rehabilitation Medicine, The Affiliated Jianhu Hospital of Xinglin College, Nantong University, Yancheng, 224700 China

**Keywords:** Stress hyperglycemia ratio, Delirium, Critically ill adults, MIMIC‑IV, Endocrine system and metabolic diseases, Risk factors, Disorders of consciousness

## Abstract

Delirium is a common neuropsychiatric complication in intensive care unit (ICU) patients and is associated with poor outcomes. This retrospective cohort study analyzed 2946 ICU patients from the MIMIC-IV database to examine whether the stress hyperglycemia ratio (SHR) is associated with delirium. SHR was classified into quartiles: Q1 (≤ 0.85), Q2 (0.86–1.01), Q3 (1.02–1.24), and Q4 (≥ 1.25). Delirium was assessed by confusion assessment method for the ICU (CAM-ICU). Logistic regression analyses with sequential adjustment for covariates were conducted across four models. Dose–response relationships were examined using restricted cubic splines (RCS), and subgroup analyses were performed to test interactions. After the first 24 h of being in the ICU, 21% (n = 619) of patients developed delirium for the first time. Higher SHR was associated with greater delirium risk in the unadjusted and partially adjusted models. After full adjustment, the continuous estimate narrowly missed statistical significance (Model 4: OR = 1.24, 95% CI 0.99–1.56; *p* = 0.057). When SHR was divided into quartiles, compared to Q1, patients in Q3 (OR = 1.58, 95% CI 1.14–2.2) and Q4 (OR = 1.59, 95% CI 1.15–2.21) had higher risk, with a significant trend (*p* for trend < 0.001). RCS analysis indicated a nonlinear relationship, and threshold-effect analysis identified an inflection point at SHR = 1.192. Subgroup analyses detected no significant interactions, with the positive association between SHR and delirium risk consistent across all subgroups. Four sensitivity analyses further supported the robustness of the SHR–delirium association. Overall, SHR showed a positive, nonlinear relationship with delirium in ICU patients, with an inflection point at 1.192, and may serve as a pragmatic predictor to inform delirium prevention in critical illness.

**Supplementary Information:**

The online version contains supplementary material available at 10.1038/s41598-026-40380-2.

## Introduction

Delirium, an acute syndrome of brain dysfunction characterized by fluctuating disturbances in attention, awareness, and cognition, is a pervasive complication in the intensive care unit (ICU), affecting 30% to 80% of patients^1^. It is associated with a cascade of adverse outcomes, including prolonged hospitalization, increased healthcare costs, greater morbidity and mortality, and long-term cognitive impairment after discharge^2,3^. While its pathophysiology is not fully elucidated, it is understood to be a multifactorial process. Key proposed mechanisms include profound neurotransmitter dysregulation (marked by decreased availability of acetylcholine and/or melatonin, alongside excessive release of dopamine, norepinephrine, and glutamate), pervasive neuroinflammatory processes, and the disruption of neural network connectivity, which may arise from imbalances in underlying neuroanatomical substrates^4,5^. Despite its high prevalence and detrimental impact, reliable and practical tools for early risk stratification remain scarce.

Previous studies have demonstrated that hyperglycemia correlates with negative outcomes in critically ill patients, including delirium^6^ and elevated mortality^7–10^. Conventionally, this risk has often been attributed to preexisting or poorly controlled diabetes. However, in the critically ill population, hyperglycemia on admission is a heterogeneous state, potentially reflecting pre-existing chronic dysglycemia stemming from poorly controlled diabetes, an acute hyperglycemic response to severe illness (stress hyperglycemia), or a combination of the two^11^. Crucially, emerging evidence indicates that stress hyperglycemia, rather than chronic hyperglycemia alone, confers a substantially greater risk^12,13^. To disentangle chronic hyperglycemia from acute stress hyperglycemia, Roberts et al.^11^ introduced the stress hyperglycemia ratio (SHR), defined as the ratio of admission glucose to the estimated average glucose, which is calculated from glycated hemoglobin (HbA1c) levels. This metric is therefore able to more accurately quantify the relative glycemic elevation attributable to acute stress, and it has demonstrated significant prognostic value in cardiovascular disease, stroke, and severe infections^14–18^.

Beyond its association with general critical care outcomes, SHR in neurological complications, particularly delirium, is an area of increasing investigation. However, the available evidence is conflicting. Some studies have identified elevated SHR as an independent risk factor for delirium^16,19^. In contrast, other research points to a U-shaped relationship, where both low and high SHR are associated with an increased delirium risk^20^. Complicating the picture further, a study shows stress hyperglycaemia is beneficial in an acute life-threatening situation^21^. This leaves the precise relationship between SHR and delirium unresolved.

From a pathophysiological standpoint, stress hyperglycaemia is characterized by the excessive release of catecholamines, such as dopamine and norepinephrine, which can impair neuronal function through pathways including oxidative stress, neuroinflammation, endothelial dysfunction, and dysregulation of cerebral blood flow. Given that these mechanisms are also critically implicated in the pathogenesis of delirium, we therefore have reason to believe that a significant association exists between the magnitude of the SHR and the development of delirium in critically ill patients. Based on this, we speculated that SHR could be linked to an increased risk of delirium among patients admitted to the ICU. This study aimed to examine the relationship between SHR and the risk of delirium in ICU patients, as well as to evaluate the potential clinical value of SHR as an indicator for predicting delirium.

This study sought to investigate the association between SHR and the risk of delirium in ICU patients, in addition to assessing the potential clinical utility of SHR as a predictive indicator for delirium.

## Methods

### Data source

A retrospective study was conducted using data from the Medical Information Mart for Intensive Care (MIMIC-IV, version 3.1), an open-access database^22^. The database has received ethical approval from both the Massachusetts Institute of Technology and the Beth Israel Deaconess Medical Center (BIDMC, Boston, MA, USA). Access to the data was granted to Chong Wang following completion of the National Institutes of Health’s “Protecting Human Research Participants” course (Record ID: 54909339). All patient information was de-identified, and informed consent was not required as determined by the BIDMC Ethics Committee. This study was conducted in accordance with the STROBE guidelines and the Declaration of Helsinki.

### Study population

**Fig. 1:**
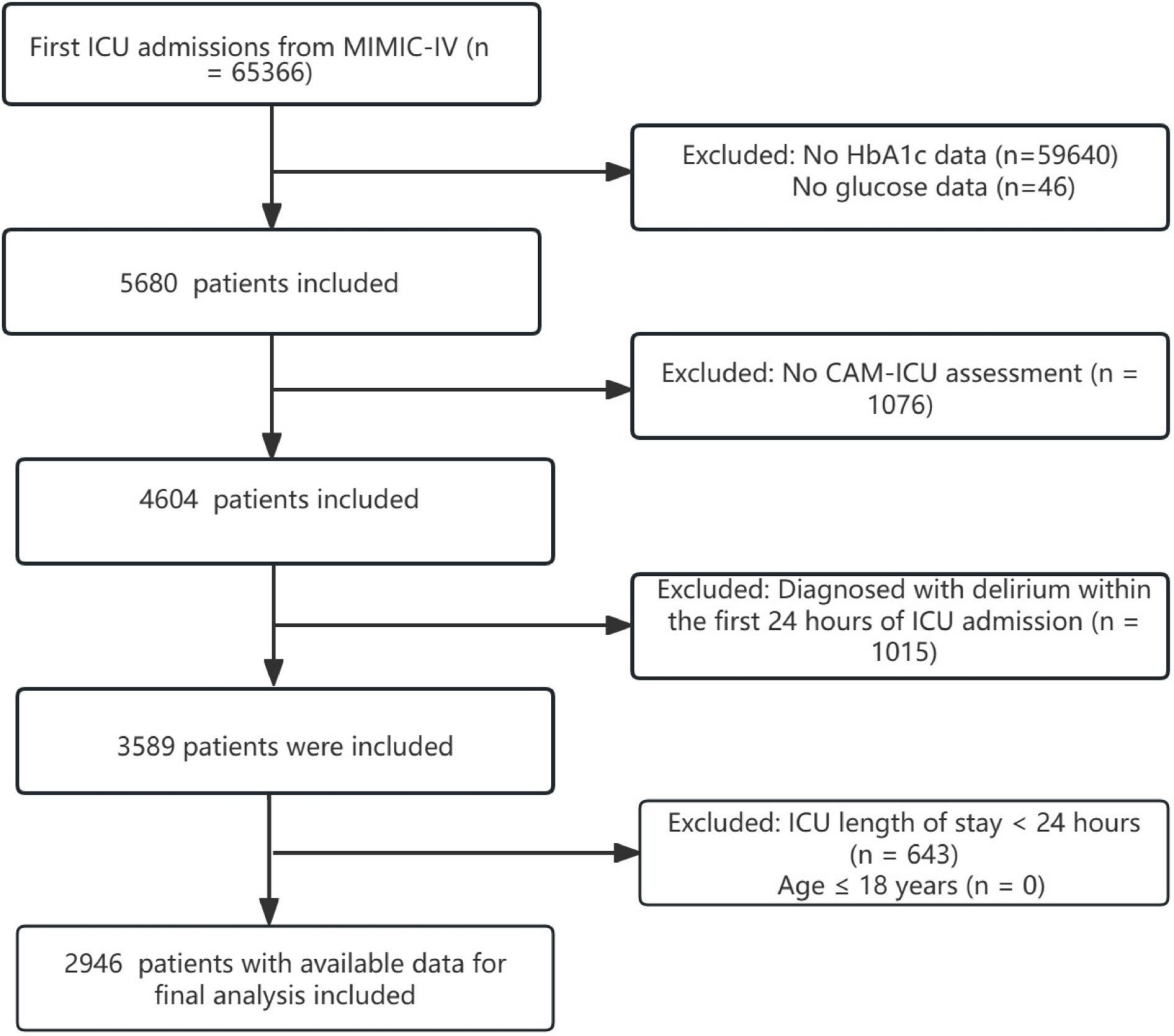
A flow chart of study patients.

To mitigate potential selection bias and confounding arising from repeat admissions, this analysis was exclusively restricted to the first ICU admission for each adult patient in the MIMIC-IV database^15,23^. Patients were excluded if they (1) lacked glucose or HbA1c measurements, (2) did not have CAM-ICU assessments, (3) were diagnosed with delirium within the first 24 h of ICU admission, or (4) had ICU stays shorter than 24 h. Among the 65,366 individuals initially admitted, a total of 2946 participants who met all inclusion criteria were ultimately analyzed (Fig. 1).

### Data collection

Navicat Premium (version 16.3.2) was used to extract baseline patient characteristics from the MIMIC-IV database via Structured Query Language (SQL) queries. Collected data included demographics (age, sex, race); vital signs (heart rate, respiratory rate, temperature, blood pressure, and oxygen saturation); and laboratory variables (white blood cell count, hemoglobin, platelets, sodium, calcium, blood urea nitrogen, creatinine, blood glucose, and HbA1c). We also recorded severity scores (SOFA, APS III, SAPS II); neurologic status assessed by the Glasgow Coma Scale (GCS); comorbidities (hypertension, diabetes, congestive heart failure, chronic pulmonary disease, cerebrovascular disease, sepsis, renal disease, liver disease, and malignant cancer); clinical outcomes (delirium, ICU and hospital lengths of stay, and mortality); and medications (sedatives and corticosteroids). To assess the impact of inflammatory markers, neutrophil and lymphocyte counts were obtained to calculate the neutrophil-to-lymphocyte ratio (NLR) and platelet-to-lymphocyte ratio (PLR). Comorbidities were identified using ICD codes from both ICD-9 and ICD-10. Cerebrovascular disease (a binary covariate) was defined using relevant ICD-9-CM (430-438, 362.34) and ICD-10-CM (G45-G46, I60-I69, H34.0) diagnosis codes, based on the Charlson Comorbidity Index coding framework. This definition encompassed ischemic and hemorrhagic stroke, transient ischemic attack, cerebral arterial occlusion, and related conditions. Sedative medications included dexmedetomidine, diazepam, etomidate, ketamine, lorazepam, midazolam, propofol, and thiopental sodium. Corticosteroid medications included hydrocortisone, methylprednisolone, prednisone, prednisolone, and dexamethasone. All laboratory parameters, severity scores, and GCS were the first recorded values within the first 24 h of ICU admission; medications were recorded on the first day. Variables were extracted using SQL queries provided by the official MIMIC GitHub repository (https://github.com/MIT-LCP/mimic-code/tree/main/mimic-iv/concepts/firstday).

### SHR score definition and grouping

SHR was calculated as the ratio of the initial blood glucose measured at ICU admission to the estimated glucose value. Both the venous blood glucose and HbA1c were the first measurements obtained within 24 h of ICU admission^15^. The estimated glucose was determined from HbA1c using the formula (mg/dL) = 28.7 × HbA1c (%) − 46.7. According to SHR quartiles, participants were assigned into four groups, designated as Q1 (SHR ≤ 0.85), Q2 (0.86–1.01), Q3 (1.02–1.24), and Q4 (≥ 1.25).

### Delirium assessment

Delirium status was assessed using the CAM-ICU, a well-validated instrument with high sensitivity and specificity in ICU settings, enabling reliable detection^24,25^. CAM-ICU screening was nurse-led and typically performed once or multiple times per day^1^. CAM-ICU assessments were performed in patients with a Richmond Agitation-Sedation Scale (RASS) score ≥  − 3; those with lower scores (− 4 or − 5) were considered too deeply sedated and were recorded as “not applicable” for that assessment period. Delirium was considered present when the following criteria were met: (1) an acute change or a fluctuating course in mental status and (2) inattention, plus either (3) disorganized thinking or (4) an altered level of consciousness^26^. Any positive CAM-ICU at any time indicated delirium. For patients assessed for delirium, delirium duration was defined as the number of ICU calendar days with at least one positive CAM-ICU assessment^27,28^. Patients with delirium on the day of admission were excluded because the episode may have occurred at or prior to admission. Accordingly, the analysis was restricted to delirium occurring more than 24 h after admission.

### Statistical analysis

Continuous variables were presented as means with standard deviations or as medians with interquartile ranges. Categorical variables were summarized as frequencies and percentages. Differences between delirium and no-delirium groups and across SHR quartiles were assessed using the t-test, one-way ANOVA, the Kruskal–Wallis test, or the chi-square test, as appropriate.

Covariate selection for the multivariable models followed a prespecified strategy integrating statistical and clinical considerations. Variables were included if they met at least one of the following criteria: (1) clinical relevance supported by established literature and pathophysiological rationale; (2) a univariable association with the outcome (*p* < 0.10) (Supplementary Table 1); or (3) a > 10% change in the primary exposure effect estimate upon entry to the model. This approach was intended to minimize confounding while preserving model parsimony. Multicollinearity was assessed using the variance inflation factor (VIF); values ≥ 5 indicated multicollinearity. Variables exhibiting multicollinearity were excluded from the final model (Supplementary Table 2). To maximize statistical power and minimize bias, missing covariates were imputed using multiple imputation by chained equations with five imputations. Covariate missingness is shown in Supplementary Table 3.

Associations between SHR and delirium were examined using univariable and multivariable logistic regression across four models: Model 1: unadjusted. Model 2: adjusted for age, sex, and race. Model 3: adjusted for model 2 + heart rate, respiratory rate, temperature, systolic blood pressure, diastolic blood pressure, oxygen saturation, white blood cell count, hemoglobin, platelet count, sodium, calcium, blood urea nitrogen, creatinine, hypertension, diabetes, congestive heart failure, chronic pulmonary disease, cerebrovascular disease, sepsis, renal disease, liver disease, malignant cancer, platelet-to-lymphocyte ratio, and neutrophil-to-lymphocyte ratio. Model 4: adjusted for model 3 + Acute Physiology Score III, Simplified Acute Physiology Score II, Glasgow Coma Scale, corticosteroid, and sedative use. Dose–response relationships were evaluated using restricted cubic splines. Subgroup analyses were performed by age (< 65 vs ≥ 65 years), sex (female vs male), diabetes (no vs yes), congestive heart failure (no vs yes), cerebrovascular disease (no vs yes), sepsis (no vs yes), GCS (< 8 vs ≥ 8), SAPS II (< 45 vs ≥ 45), and sedation use (no vs yes), with interaction assessed by *P* values; results were presented as forest plots. Sensitivity analyses included repeating the primary analyses in the complete-case dataset, excluding participants with DM, excluding those with ICU LOS > 30 days, and excluding those with SHR < 1.192. ROC analysis evaluated whether SHR provided better discrimination for delirium than glucose. All analyses were performed using R version 4.0.3 (The R Foundation) and Free Statistics software version 2.1 (Beijing, China). Statistical significance was defined as a two-tailed *P*-value < 0.05.

## Results

### Baseline characteristics of the study population

We analyzed 2946 ICU patients (mean age 64.3 ± 16.0 years; 57.6% male). The mean SHR was 1.11 ± 0.46; delirium occurred in 21.0% of patients. Among patients with delirium, 38.6%, 19.5%, 11.6%, and 30.2% experienced 1, 2, 3, and ≥ 4 delirium days, respectively; The median numbers of delirium, non-delirium, and not-assessable ICU days were 2.0 (1.0–4.0), 3.0 (1.0–5.0), and 1.0 (1.0–4.0), respectively.

Compared with patients without delirium, those with delirium were older; had higher heart rate, respiratory rate, and body temperature but lower systolic and diastolic blood pressure; had a lower GCS score but higher APSIII, SAPSII, and SOFA scores; had a higher prevalence of congestive heart failure, chronic pulmonary disease, cerebrovascular disease, sepsis, renal and liver disease, and malignancy; had higher WBC, BUN, creatinine, glucose, NLR, and SHR but lower hemoglobin, platelet count, calcium, and HbA1c levels; were more likely to receive sedatives and corticosteroids; and had longer ICU and hospital stays with higher ICU and in-hospital mortality (Table 1). Baseline characteristics by SHR quartile are presented in Supplementary Table 4.

**Table 1:** Baseline characteristics of the study participants.

Variables	Total (n = 2946)	Without delirium (n = 2327)	With delirium (n = 619)	*P*
Age, years	64.29 ± 16.03	63.37 ± 16.43	67.76 ± 13.90	< 0.001
Sex				0.381
Female	1249 (42.4)	977 (42)	272 (43.9)	
Male	1697 (57.6)	1350 (58)	347 (56.1)	
Race				0.004
White	1616 (54.9)	1303 (56)	313 (50.6)	
Black/Africa American	316 (10.7)	258 (11.1)	58 (9.4)	
Other	1014 (34.4)	766 (32.9)	248 (40.1)	
Vital signs				
Heart rate, beats/min	80.81 ± 15.60	80.02 ± 15.43	83.79 ± 15.87	< 0.001
Respiratory rate, breaths/min	19.09 ± 3.40	18.76 ± 3.23	20.32 ± 3.74	< 0.001
Temperature, °C	36.88 ± 0.36	36.85 ± 0.29	36.96 ± 0.54	< 0.001
Systolic blood pressure, mmHg	126.38 ± 18.31	127.01 ± 18.08	124.06 ± 18.98	< 0.001
Diastolic blood pressure, mmHg	70.77 ± 12.58	71.64 ± 12.46	67.48 ± 12.58	< 0.001
SpO_2_, %	96.53 ± 1.84	96.46 ± 1.80	96.80 ± 1.97	< 0.001
Scoring systems				
GCS	12.87 ± 3.12	13.64 ± 2.35	10.01 ± 3.88	< 0.001
APSⅢ	39.98 ± 19.94	35.70 ± 16.09	56.07 ± 24.34	< 0.001
SAPSⅡ	30.50 ± 11.68	28.60 ± 10.77	37.67 ± 12.17	< 0.001
SOFA	3.0 (1.0, 5.0)	2.0 (1.0, 4.0)	5.0 (3.0, 8.0)	< 0.001
Comorbidities, n(%)				
Hypertension	520 (17.7)	403 (17.3)	117 (18.9)	0.359
Diabetes	1208 (41.0)	946 (40.7)	262 (42.3)	0.452
Myocardial infarct	806 (27.4)	628 (27)	178 (28.8)	0.38
Congestive heart failure	824 (28.0)	586 (25.2)	238 (38.4)	< 0.001
Chronic pulmonary disease	512 (17.4)	375 (16.1)	137 (22.1)	< 0.001
Cerebrovascular disease	1501 (51.0)	1156 (49.7)	345 (55.7)	0.007
Sepsis	932 (31.6)	515 (22.1)	417 (67.4)	< 0.001
Renal disease	498 (16.9)	371 (15.9)	127 (20.5)	0.007
Liver disease	222 (7.5)	142 (6.1)	80 (12.9)	< 0.001
Malignant cancer	168 (5.7)	121 (5.2)	47 (7.6)	0.022
Laboratory tests				
WBC (10⁹/L)	9.65 ± 4.24	9.34 ± 3.92	10.83 ± 5.12	< 0.001
Hemoglobin (g/dL)	11.69 ± 2.29	11.84 ± 2.23	11.10 ± 2.41	< 0.001
Platelet (10⁹/L)	207.26 ± 77.87	209.17 ± 77.01	199.98 ± 80.83	0.009
Sodium (mmol/L)	136.95 ± 4.68	137.00 ± 4.50	136.76 ± 5.29	0.271
Potassium (mmol/L)	3.86 ± 0.49	3.87 ± 0.48	3.83 ± 0.54	0.119
Calcium (mg/dL)	8.52 ± 0.76	8.58 ± 0.73	8.27 ± 0.86	< 0.001
Chloride (mmol/L)	101.17 ± 5.49	101.16 ± 5.32	101.19 ± 6.10	0.924
BUN (mg/dL)	15.0 (11.0, 22.0)	15.0 (11.0, 21.0)	17.0 (13.0, 26.0)	< 0.001
Creatinine (mg/dL)	0.9 (0.7, 1.1)	0.8 (0.7, 1.1)	0.9 (0.7, 1.3)	< 0.001
Glucose (mg/dL)	156.90 ± 96.83	153.76 ± 97.63	168.71 ± 92.92	< 0.001
NLR	5.26 (2.91, 8.88)	4.97 (2.80, 8.20)	6.65 (3.56, 11.86)	< 0.001
PLR	165.57 (104.64, 286.78)	163.71 (106.39, 285.49)	171.52 (97.87, 295.41)	0.672
HbA1c(%)	6.63 ± 2.10	6.68 ± 2.19	6.44 ± 1.70	0.012
SHR	1.11 ± 0.46	1.08 ± 0.44	1.24 ± 0.53	< 0.001
Treatments and clinical outcomes				
Sedative medications, n(%)	1131 (38.4)	747 (32.1)	384 (62)	< 0.001
Corticosteroids, n(%)	80 (2.7)	47 (2)	33 (5.3)	< 0.001
ICU length of stay, days	2.7 (1.7, 4.9)	2.2 (1.6, 3.7)	6.6 (3.9, 12.3)	< 0.001
Hospital length of stay,days	6.4 (3.7, 11.5)	5.3 (3.2, 8.7)	14.8 (8.8, 23.7)	< 0.001
ICU mortality, n(%)	129 (4.4)	62 (2.7)	67 (10.8)	< 0.001
Hospital mortality, n (%)	192 (6.5)	89 (3.8)	103 (16.6)	< 0.001

GCS, Glasgow Coma Scale; APS III, Acute Physiology Score III; SAPS II, Simplified Acute Physiology Score II; SOFA, Sequential Organ Failure Assessment; WBC, White Blood Cell count; BUN, Blood Urea Nitrogen; NLR, Neutrophil to Lymphocyte Ratio; PLR, Platelet to Lymphocyte Ratio; HbA1c, Glycated Hemoglobin; SHR, Stress Hyperglycemia Ratio.

### Association of SHR with risk of delirium

Multivariable logistic regression (Table 2) assessed the association between SHR and delirium. When modeled as a continuous variable, SHR was positively associated with delirium in Models 1–3 (Model 1: OR = 1.90, 95% CI 1.58–2.27, *p* < 0.001; Model 2: OR = 1.92, 95% CI 1.61–2.30, *p* < 0.001; Model 3: OR = 1.40, 95% CI 1.14–1.73, *p* = 0.002), but the association attenuated and was borderline in the fully adjusted model (Model 4: OR = 1.24, 95% CI 0.99–1.56; *p* = 0.057). When SHR was divided into quartiles, compared to Q1, the risk of delirium in Q3 (OR = 1.58, 95% CI 1.14–2.20, *p* = 0.006) and Q4 (OR = 1.59, 95% CI 1.15–2.21, *p* = 0.006) increased significantly after full adjustment. Trend analysis confirmed a significant dose–response relationship as SHR quartile increased (*p* for trend = 0.001).

**Table 2:** Multivariable logistical regression for SHR and delirium.

Variable	n(total)	n (%)	Model1		Model2		Model3		Model4	
			OR (95%CI)	*P*	OR (95%CI)	*P*	OR (95%CI)	*P*	OR (95%CI)	*P*
SHR	2946	619 (21)	1.9 (1.58 ~ 2.27)	< 0.001	1.92 (1.61 ~ 2.3)	< 0.001	1.4 (1.14 ~ 1.73)	0.002	1.24 (0.99 ~ 1.56)	0.057
Q1(≤ 0.85)	737	107 (14.5)	1(Ref)		1(Ref)		1(Ref)		1(Ref)	
Q2(0.86–1.01)	720	111 (15.4)	1.07 (0.8 ~ 1.43)	0.631	1.06 (0.79 ~ 1.42)	0.689	1.1 (0.79 ~ 1.52)	0.572	1.03 (0.73 ~ 1.46)	0.852
Q3(1.02–1.24)	742	173 (23.3)	1.79 (1.37 ~ 2.34)	< 0.001	1.75 (1.33 ~ 2.28)	< 0.001	1.6 (1.18 ~ 2.17)	0.003	1.58 (1.14 ~ 2.2)	0.006
Q4 (≥ 1.25)	747	228 (30.5)	2.59 (2 ~ 3.35)	< 0.001	2.55 (1.97 ~ 3.31)	< 0.001	1.79 (1.32 ~ 2.42)	< 0.001	1.59 (1.15 ~ 2.21)	0.006
Trend test	2946			< 0.001		< 0.001		< 0.001		0.001

Model1: unadjusted.

Model2: adjusted for age, sex, and race.

Model3: adjusted for model2 + heart rate, respiratory rate, temperature, systolic blood pressure, diastolic blood pressure, oxygen saturation, white blood cell count, hemoglobin, platelet count, sodium, calcium, blood urea nitrogen, creatinine, hypertension, diabetes, congestive heart failure, chronic pulmonary disease, cerebrovascular disease, sepsis, renal disease, liver disease, malignant cancer, platelet-to-lymphocyte ratio, and neutrophil-to-lymphocyte ratio.

Model4: adjusted for model3 + Acute Physiology Score III, Simplified Acute Physiology Score II, Glasgow Coma Scale, corticosteroid and sedative use.

### Analysis of RCS curves and threshold effects of SHR and delirium

**Fig. 2:**
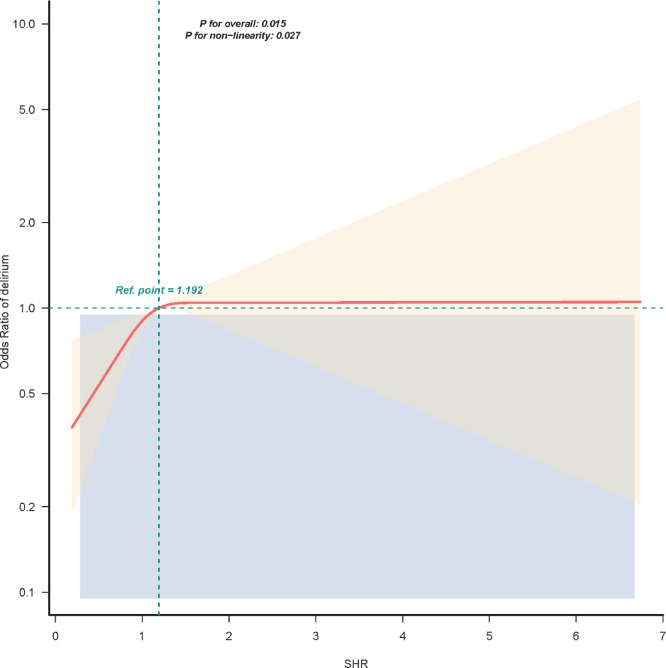
Association between SHR and delirium odds ratio. The solid red line represents the fitted curve, with pink bars denoting 95% confidence intervals. Data were adjusted for age, sex, race, heart rate, respiratory rate, temperature, systolic blood pressure, diastolic blood pressure, oxygen saturation, white blood cell count, hemoglobin, platelet count, sodium, calcium, blood urea nitrogen, creatinine, hypertension, diabetes, congestive heart failure, chronic pulmonary disease, cerebrovascular disease, sepsis, renal disease, liver disease, malignant cancer, platelet-to-lymphocyte ratio, and neutrophil-to-lymphocyte ratio, Acute Physiology Score III, Simplified Acute Physiology Score II, Glasgow Coma Scale, corticosteroid and sedative use.

RCS analysis demonstrated a nonlinear association between SHR and the odds of delirium (*p* for nonlinearity = 0.027; Fig. 2). Threshold effect analysis identified an inflection point at SHR = 1.192. Below this inflection (SHR < 1.192), higher SHR was associated with greater odds of delirium (OR 3.21, 95% CI 1.40–7.33, *p* = 0.006). At or above the inflection (SHR ≥ 1.192), the association was not statistically significant (OR 1.07, 95% CI 0.77–1.49, *p* = 0.684), indicating that once SHR exceeds 1.192, further increases do not confer additional delirium risk (Table 3).

**Table 3:** Threshold effect analysis of the relationship of SHR and delirium.

SHR	Adjusted model	
	OR (95%CI)	*P*
< 1.192	3.21 (1.40–7.33)	0.006
≥ 1.192	1.07 (0.77–1.49)	0.684
Likelihood ratio test		0.011

Data were adjusted for age, sex, race, heart rate, respiratory rate, temperature, systolic blood pressure, diastolic blood pressure, oxygen saturation, white blood cell count, hemoglobin, platelet count, sodium, calcium, blood urea nitrogen, creatinine, hypertension, diabetes, congestive heart failure, chronic pulmonary disease, cerebrovascular disease, sepsis, renal disease, liver disease, malignant cancer, platelet-to-lymphocyte ratio, and neutrophil-to-lymphocyte ratio, Acute Physiology Score III, Simplified Acute Physiology Score II, Glasgow Coma Scale, corticosteroid and sedative use.

### Subgroup analysis

**Fig. 3:**
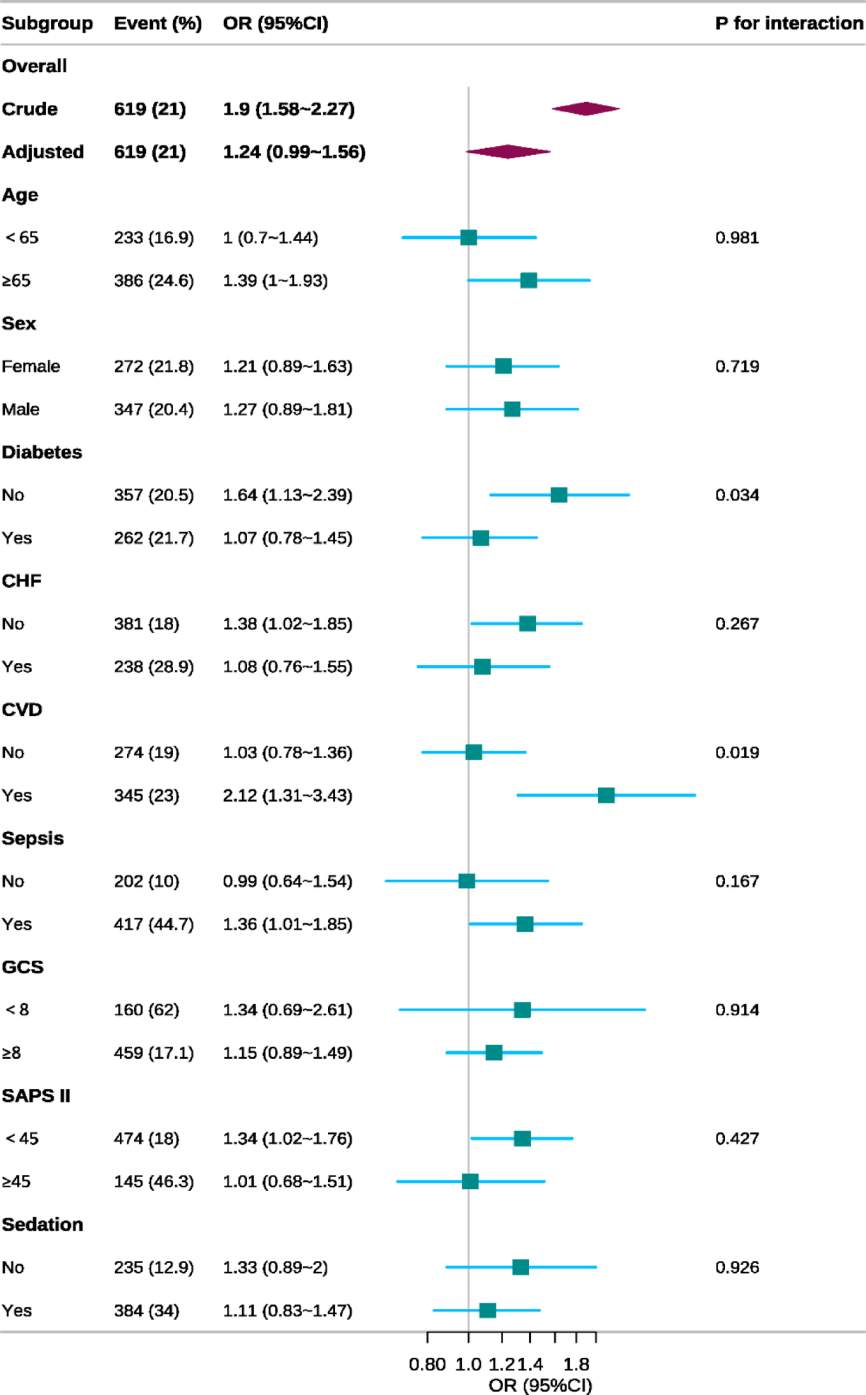
Subgroup analyses of the association between SHR and delirium according to basic features. Adjusted for age, sex, race, heart rate, respiratory rate, temperature, systolic blood pressure, diastolic blood pressure, oxygen saturation, white blood cell count, hemoglobin, platelet count, sodium, calcium, blood urea nitrogen, creatinine, hypertension, diabetes, congestive heart failure, chronic pulmonary disease, cerebrovascular disease, sepsis, renal disease, liver disease, malignant cancer, platelet-to-lymphocyte ratio, and neutrophil-to-lymphocyte ratio, Acute Physiology Score III, Simplified Acute Physiology Score II, Glasgow Coma Scale, corticosteroid and sedative use.

We conducted subgroup analyses to assess the robustness of the association between SHR and the odds of delirium (Fig. 3). Interactions by diabetes (*p* for interaction = 0.034) and CVD (*p* for interaction = 0.019) were nominally significant but did not remain significant after correction for multiple comparisons (Bonferroni-adjusted threshold ≈ 0.0056). No significant interactions were observed in the other subgroups, and the positive association between SHR and delirium was consistent across strata.

### Sensitivity analysis

To evaluate the robustness of the findings, we performed four sensitivity analyses: (1) repeated the primary analyses using the original dataset without multiple imputation (Supplementary Table 5); (2) excluded participants with diabetes (Supplementary Table 6); (3) excluded participants with ICU length of stay > 30 days (Supplementary Table 7); and (4) excluded participants with SHR > 1.192 (Supplementary Table 8). The association between SHR and delirium remained robust across all analyses.

### ROC analysis

**Fig. 4:**
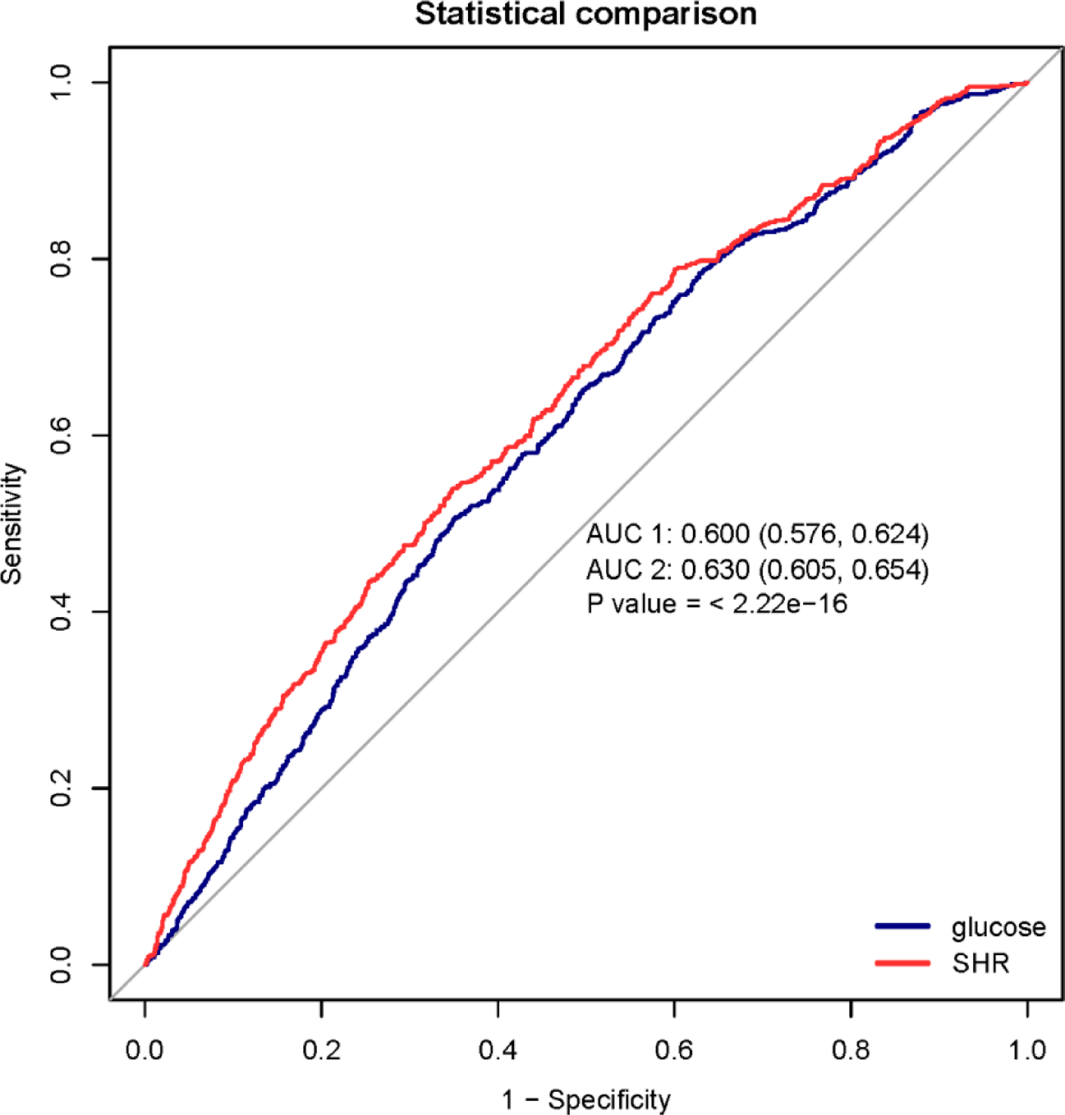
Receiver operating characteristic (ROC) curves for delirium prediction models based on SHR and glucose. AUC, area under the curve.

ROC analysis showed that SHR outperformed glucose for predicting delirium (Fig. 4). In models including sex, age, and diabetes, the AUCs were 0.630 for SHR and 0.600 for glucose (*p* < 0.001).

## Discussion

In this retrospective MIMIC‑IV cohort, higher admission SHR was independently associated with greater odds of delirium, and the association remained robust after adjustment for potential confounders. RCS analysis indicated a nonlinear relationship, and threshold-effect analysis identified an inflection point at SHR = 1.192. Subgroup analyses detected no significant interactions, with the positive association between SHR and delirium risk consistent across all subgroups. Four sensitivity analyses further supported the robustness of the SHR–delirium association. ROC analysis showed that SHR outperformed glucose in predicting delirium.

Dysglycemia in critical illness exerts complex effects on brain function and is linked to adverse neurologic outcomes^29–35^. Prior studies on glycemic status and delirium have yielded inconsistent findings: some report higher delirium risk in type 2 diabetes, whereas others suggest that dynamic metrics such as mean blood glucose or glycemic lability index are more predictive^36,37^. This inconsistency likely reflects failure to distinguish acute stress-induced hyperglycemia from chronic dysglycemia. The present study provides important evidence linking SHR to delirium risk in ICU patients.

SHR and delirium likely share overlapping pathophysiological pathways in critical illness. An elevated SHR often reflects intensified systemic inflammation and metabolic derangements that impair brain function. Accordingly, the link between higher SHR and delirium is probably multifactorial, encompassing neuroendocrine disruption, inflammatory cascades, and cellular injury. First, stress hyperglycemia signifies an acute neuroendocrine response characterized by the release of cortisol and catecholamines. These hormones can disrupt neurotransmitter systems, particularly acetylcholine and dopamine, impairing neuronal function and precipitating delirium^38,39^. Second, stress hyperglycemia exacerbates systemic inflammation, a key contributor to delirium. It promotes the release of pro-inflammatory cytokines such as IL-1β, TNF-α, and IL-6^40,41^. Finally, at the cellular level, stress hyperglycemia may induce neuronal damage by increasing reactive oxygen species production, which leads to oxidative stress, mitochondrial dysfunction, and compromised blood–brain barrier integrity^16,42^. Furthermore, it can promote insulin resistance, impairing cerebral glucose utilization and leading to bioenergetic failure, thereby increasing vulnerability to delirium^43^.

In this study, the association between SHR and delirium was pronounced among non-diabetic patients. Several factors may account for this divergence. First, in diabetes, poor glycemic control and elevated HbA1c increase baseline glycemia, producing a relatively smaller SHR. Second, in patients without diabetes, stress hyperglycemia during critical illness signals a sharp disruption of metabolic homeostasis that may cause more direct organ injury, including to the brain. Third, chronic hyperglycemia in diabetes may engender compensatory/adaptive changes that confer partial tolerance to acute glycemic fluctuations^44^. Consequently, the same degree of stress hyperglycemia may impose greater relative physiological injury in metabolically unadapted individuals. Consistent with this interpretation, new-onset in-hospital hyperglycemia is associated with higher mortality^45^, likely reflecting a more intense systemic stress response.

In our study, RCS analysis revealed a nonlinear association between SHR and the risk of delirium. One prior study suggested a U-shaped relationship^22^, but it included only 487 geriatric ward patients, whose disease severity likely differs from our critically ill cohort^20^. Threshold-effect analysis identified an inflection point at SHR = 1.192; at or above this threshold, further increases in SHR did not confer additional delirium risk. Whether higher SHR levels entail other adverse risks warrants further investigation. Subgroup analyses show the positive association between SHR and delirium risk is consistent across all subgroups, underscoring the generalizability of SHR as a key risk indicator. Sensitivity analyses further supported the robustness of this association. ROC analysis showed that SHR outperformed glucose in predicting delirium, supporting the pragmatic utility of SHR for risk stratification.

For critically ill patients, elevated SHR may signal an increased risk of delirium. Once this risk is recognized, clinicians should promptly heighten risk awareness, reassess the necessity of potentially deliriogenic medications, and implement early prevention measures—including spontaneous awakening, standardized delirium monitoring and management, early mobility, and family engagement—to reduce delirium and its potential harms^46^. As an early, easily obtainable, and low-cost indicator, SHR may have a practical role in the prevention and management of delirium in critically ill patients.

There are some limitations to this investigation. First, as a retrospective analysis, causality between the SHR and the development of delirium cannot be inferred. Second, although multivariate adjustments were made, the presence of unmeasured confounders cannot be excluded. Third, the investigation was conducted at a single center, so external validity should be further evaluated in multicenter cohorts. Fourth, SHR was calculated solely from the first blood glucose and hemoglobin A1c measurements at ICU admission, precluding assessment of glucose dynamics and variability—factors that may also be relevant. Finally, delirium subtypes (hyperactive, hypoactive, or mixed) were not distinguished in this cohort, although different subtypes may have distinct pathophysiological backgrounds and risk profiles.

Future research should prioritize prospective studies to validate the predictive value of SHR for delirium. Moreover, it is essential to clarify the predictive utility of SHR among different delirium subtypes.

## Conclusion

Admission SHR showed a positive, nonlinear association with delirium in ICU patients, with an inflection point at SHR = 1.192. Although the magnitude of the association varied among clinical subgroups, an overall positive trend remained across all subgroups. SHR may be a pragmatic predictor for delirium prevention in critically ill patients.

## Supplementary Information

Below is the link to the electronic supplementary material.

Supplementary Material 1

## Data Availability

The data used in this study were sourced from the Medical Information Mart for Intensive Care IV (MIMIC-IV, https://mimic-iv.mit.edu). Access is limited to authorized users who complete the necessary training and agree to the data use terms. Requests for dataset access can be made through PhysioNet (https://physionet.org/content/mimiciv/3.1/).

## Abbreviations

ICU: Intensive care unit
SHR: Stress hyperglycemia ratio
AMI: Acute myocardial infarction
SBP: Systolic blood pressure
DBP: Diastolic blood pressure
RR: Respiratory rate
HR: Heart rate
SpO2: Percutaneous oxygen saturation
SAPS II: Simplified acute physiology score II
GCS: Glasgow coma score
APS Ⅲ: Acute physiology and chronic health evaluation Ⅲ
CPD: Chronic pulmonary disease
WBC: White blood cell
BUN: Blood urea nitrogen
NLR: Neutrophil-to-lymphocyte ratio
PLR: Platelet-to-lymphocyte ratio
LMR: Lymphocyte-to-monocyte ratio
CRP: C-reactive protein
