# Supplementary material for: Association between stress hyperglycemia ratio and ICU delirium among critically ill adults in MIMIC-IV
Source: Sci Rep. 2026 Feb 17;16:9411. doi: 10.1038/s41598-026-40380-2 (PMC13002884; doi:10.1038/s41598-026-40380-2)
Supplement: Supplementary file 1 — Supplementary Material 1 [file 41598_2026_40380_MOESM1_ESM.doc]

Submission ID 6ea4764f-91ab-46ca-b83a-6b37e9560882

Title of Manuscript: Association of stress hyperglycaemic ratio with risk of delirium in ICU patients: a study based on the MIMIC-IV database

Title of revised manuscript: Association between stress hyperglycemia ratio and ICU delirium among critically ill adults in MIMIC IV

**Association between stress hyperglycemia ratio and ICU delirium among critically ill adults in MIMIC‑IV**

Chong Wang*, Lili Lv, Rongrong Ma, Haiyan Dong

Department of Rehabilitation Medicine, The Affiliated Jianhu Hospital of Xinglin College, Nantong University, 224700, Yancheng, China.

Corresponding author. Chong Wang: E-mail: [wangchong@ntu.edu.cn](mailto:wangchong@ntu.edu.cn)

**Supplementary files**

**Supplementary Table 1** Univariate Analysis with P-Value Less Than 0.1

**Supplementary Table 2** Results of Covariate Selection: Effect Size Change Exceeding 10% and VIF < 5

**Supplementary Table 3** Summary of missing data by variable in the study population

**Supplementary Table 4**. Baseline characteristics of the study participants across SHR quartiles

**Supplementary Table 5** Multivariable logistical regression for SHR and delirium in the nonimputed dataset

**Supplementary Table 6** Multivariable logistical regression for SHR and delirium excluded participants with diabetes

**Supplementary Table 7** Multivariable logistical regression for SHR and delirium excluded participants with ICU length of stay > 30 days

**Supplementary Table 8** Multivariable logistical regression for SHR and delirium excluded participants with SHR > 1.192

**Supplementary Table 1 Univariate Analysis with P-Value Less Than 0.1**

| Variable | OR (95%CI) | ***P* value** |
| --- | --- | --- |
| Gender,(Male vs Female) | 0.92 (0.77~1.1) | 0.381 |
| Age | 1.02 (1.01~1.02) | <0.001 |
| Race, n (%) |  | 0.675 |
| White | 1(reference) |  |
| Black/Africa American | 0.94 (0.69~1.28) |  |
| Other | 1.35 (1.12~1.63) | 0.002 |
| Heart rate | 1.02 (1.01~1.02) | <0.001 |
| Systolic blood pressure, mmHg | 0.99 (0.99~1) | 0.001 |
| Diastolic blood pressure, mmHg | 0.97 (0.97~0.98) | <0.001 |
| Respiratory rate, breaths/min | 1.13 (1.11~1.16) | <0.001 |
| Temperature, °C | 2.31 (1.78~2.99) | <0.001 |
| SpO₂, % | 1.11 (1.06~1.17) | <0.001 |
| Hemoglobin (g/dL) | 0.87 (0.84~0.9) | <0.001 |
| Platelet (10⁹/L) | 1 (1~1) | 0.009 |
| WBC (10⁹/L) | 1.08 (1.06~1.1) | <0.001 |
| BUN (mg/dL) | 1.02 (1.01~1.02) | <0.001 |
| Calcium(mg/dL) | 0.61 (0.55~0.69) | <0.001 |
| Chloride (mmol/L) | 1 (0.98~1.02) | 0.924 |
| Creatinine (mg/dL) | 1.1 (1.03~1.18) | 0.005 |
| Sodium (mmol/L) | 0.99 (0.97~1.01) | 0.271 |
| Potassium (mmol/L) | 0.87 (0.72~1.04) | 0.119 |
| Myocardial infarct,(yes to no) | 1.09 (0.9~1.33) | 0.38 |
| Congestive heart failure,(yes to no) | 1.86 (1.54~2.24) | <0.001 |
| Cerebrovascular disease,(yes to no) | 1.28 (1.07~1.52) | 0.007 |
| Chronic pulmonary disease,(yes to no) | 1.48 (1.19~1.84) | <0.001 |
| Renal_disease,(yes to no) | 1.36 (1.09~1.7) | 0.007 |
| Malignant cancer,(yes to no) | 1.5 (1.06~2.12) | 0.023 |
| Diabetes,(yes to no) | 1.07 (0.9~1.28) | 0.452 |
| Hypertension ,(yes to no) | 1.11 (0.89~1.4) | 0.359 |
| Cerebral infarction ,(yes to no) | 1.09 (0.9~1.31) | 0.383 |
| Liver disease,(yes to no) | 2.28 (1.71~3.05) | <0.001 |
| APSIII | 1.05 (1.05~1.06) | <0.001 |
| SAPSII | 1.07 (1.06~1.08) | <0.001 |
| GCS | 0.72 (0.7~0.74) | <0.001 |
| Sepsis | 7.26 (5.98~8.82) | <0.001 |
| SOFA | 1.4 (1.36~1.45) | <0.001 |
| Corticoid | 2.73 (1.73~4.3) | <0.001 |
| Sedation | 3.46 (2.87~4.16) | <0.001 |
| RDW | 1.13 (1.08~1.18) | <0.001 |
| Albumin | 0.45 (0.36~0.56) | <0.001 |
| Lymphocytes | 1.01 (0.99~1.03) | 0.286 |
| Neutrophils | 1.05 (1.03~1.07) | <0.001 |
| RA | 1.38 (1.25~1.53) | <0.001 |
| PLR | 1 (1~1) | 0.265 |
| NAR | 1.22 (1.14~1.31) | <0.001 |
| NLR | 1.02 (1.01~1.04) | <0.001 |

**Abbreviations:** WBC, white blood cell; APS III, Acute Physiology Score III; GCS, Glasgow Coma Scale; NAR, neutrophil-to-albumin ratio; NLR, neutrophil-to-lymphocyte ratio; PLR, platelet-to-lymphocyte ratio; RA, red cell distribution width-to-albumin ratio; RDW, red blood cell distribution width; SAPS II, Simplified Acute Physiology Score II; SOFA, Sequential Organ Failure Assessment.

**Supplementary Table 2** Results of Covariate Selection: Effect Size Change Exceeding 10% and VIF < 5

| Term1 | Change.percentage1 | Change.percentage2 | GVIF | DF | GVIF^(1/(2*Df)) | colinearity | select |
| --- | --- | --- | --- | --- | --- | --- | --- |
| Crude | Ref. | Ref. | 1.323 | 1 | 1.15 | 0 | Ref. |
| Sex | 0.2 | -15.7 | 1.264 | 1 | 1.124 | 0 | Yes |
| Age | 2.6 | -6.7 | 2.171 | 1 | 1.473 | 0 | No |
| Race | -0.2 | 2.7 | 1.376 | 2 | 1.083 | 0 | No |
| Heart rate | -7.4 | -20.5 | 1.829 | 1 | 1.352 | 0 | Yes |
| Systolic blood pressure | -8.5 | -0.2 | 2.096 | 1 | 1.448 | 0 | No |
| Diastolic blood pressure | -13.2 | -3.8 | 2.424 | 1 | 1.557 | 0 | Yes |
| Respiratory rate | -13.2 | 0.8 | 1.568 | 1 | 1.252 | 0 | Yes |
| Temperature | 0.5 | -2 | 1.247 | 1 | 1.117 | 0 | No |
| SpO₂ | -1.8 | -1.6 | 1.425 | 1 | 1.194 | 0 | No |
| Hemoglobin | -12 | -0.7 | 2.028 | 1 | 1.424 | 0 | Yes |
| Platelet | -1.4 | 1.9 | 1.599 | 1 | 1.265 | 0 | No |
| WBC | -18.6 | 0.1 | 2.124 | 1 | 1.458 | 0 | Yes |
| BUN | -10.9 | 0 | 2.942 | 1 | 1.715 | 0 | Yes |
| Calcium | -24 | 42.3 | 1.871 | 1 | 1.368 | 0 | Yes |
| Chloride | 3.2 | 6.1 | 3.613 | 1 | 1.901 | 0 | No |
| Creatinine | -3.3 | -0.3 | 3.02 | 1 | 1.738 | 0 | No |
| Sodium | -0.1 | 24.4 | 3.764 | 1 | 1.94 | 0 | Yes |
| Potassium | -0.4 | 5.4 | 1.453 | 1 | 1.205 | 0 | No |
| Myocardial infarct | -0.2 | -2.6 | 1.407 | 1 | 1.186 | 0 | No |
| Congestive heart failure | -9.6 | 1.8 | 1.507 | 1 | 1.227 | 0 | No |
| Cerebrovasculardisease | 12.9 | 0.9 | 2.876 | 1 | 1.696 | 0 | Yes |
| Chronic pulmonary disease | -2.7 | -0.6 | 1.112 | 1 | 1.054 | 0 | No |
| Renal disease | -3 | 2.2 | 1.582 | 1 | 1.258 | 0 | No |
| Malignant_cancer | -2.1 | 0.1 | 1.252 | 1 | 1.119 | 0 | No |
| Diabetes | -0.4 | -1.5 | 1.222 | 1 | 1.106 | 0 | No |
| Hypertension | -0.1 | -4.3 | 2.131 | 1 | 1.46 | 0 | No |
| Cerebral infarction | 4.8 | 2.1 | 2.499 | 1 | 1.581 | 0 | No |
| Liver disease | -7.7 | 4.2 | 1.234 | 1 | 1.111 | 0 | No |
| APSⅢ | -72.7 | 4.8 | 4.091 | 1 | 2.023 | 0 | Yes |
| SAPSⅡ | -43.8 | -6.7 | 3.206 | 1 | 1.791 | 0 | Yes |
| GCS | -14.2 | -50.4 | 2.695 | 1 | 1.642 | 0 | Yes |
| Sepsis3 | -43.2 | 10.4 | 1.498 | 1 | 1.224 | 0 | Yes |
| Sofa | -78.7 | 14 | 3.581 | 1 | 1.892 | 0 | Yes |
| RDW | -6.1 | -4.2 | 6.235 | 1 | 2.497 | 1 | No |
| Albumin | -13.1 | -3.3 | 9.332 | 1 | 3.055 | 1 | Yes |
| Lymphocytes | 6.4 | 0.7 | 1.255 | 1 | 1.12 | 0 | No |
| Neutrophils | -7.1 | 4.3 | 31.121 | 1 | 5.579 | 1 | No |
| Corticoid | -4.2 | 6.2 | 1.14 | 1 | 1.068 | 0 | No |
| Sedation | -15.6 | 21.4 | 1.536 | 1 | 1.239 | 0 | Yes |
| RA | -10.4 | -5.6 | 18.453 | 1 | 4.296 | 1 | Yes |
| PLR | 7.7 | 0.7 | 2.718 | 1 | 1.649 | 0 | No |
| NAR | -4.3 | 5.9 | 31.86 | 1 | 5.645 | 1 | No |
| NLR | -5.8 | 24.1 | 2.191 | 1 | 1.48 | 0 | Yes |

Abbreviations: APS III, Acute Physiology Score III; GCS, Glasgow Coma Scale; NAR, neutrophil-to-albumin ratio; NLR, neutrophil-to-lymphocyte ratio; PLR, platelet-to-lymphocyte ratio; RA, red cell distribution width-to-albumin ratio; RDW, red blood cell distribution width; SAPS II, Simplified Acute Physiology Score II; SOFA, Sequential Organ Failure Assessment.

Supplementary Table 3 Summary of missing data by variable in the study population

| Variable | Missing Count | Missing Percentage (%) |
| --- | --- | --- |
| PLR | 992 | 33.6728 |
| NLR | 988 | 33.537 |
| Calcium | 139 | 4.7183 |
| Temperature | 24 | 0.8147 |
| DBP | 12 | 0.4073 |
| SBP | 12 | 0.4073 |
| Platelets | 9 | 0.3055 |
| Respiratory rate | 9 | 0.3055 |
| Hemoglobin | 6 | 0.2037 |
| White blood cell count | 5 | 0.1697 |
| Creatinine | 1 | 0.0339 |
| Glasgow Coma Scale | 1 | 0.0339 |
| Potassium | 1 | 0.0339 |

Abbreviations: DBP, diastolic blood pressure; GCS, Glasgow Coma Scale; NLR, neutrophil-to-lymphocyte ratio; PLR, platelet-to-lymphocyte ratio; SBP, systolic blood pressure.

**Supplementary Table 4. Baseline characteristics of the study participants across SHR quartiles**

| Variables | Total (n = 2946) | Q1(≤0.85) (n = 737) | Q2(0.86-1.01) (n = 720) | Q3 (1.02-1.24) (n = 742) | Q4(≥1.25) (n = 747) | P |
| --- | --- | --- | --- | --- | --- | --- |
| **Demographic characteristics** |  |  |  |  |  |  |
| Age, years | 64.29 ± 16.03 | 63.30 ± 17.23 | 64.07 ± 16.26 | 65.28 ± 15.09 | 64.49 ± 15.43 | 0.117 |
| Sex |  |  |  |  |  | 0.389 |
| Female | 1249 (42.4) | 332 (45) | 303 (42.1) | 304 (41) | 310 (41.5) |  |
| Male | 1697 (57.6) | 405 (55) | 417 (57.9) | 438 (59) | 437 (58.5) |  |
| Race |  |  |  |  |  | < 0.001 |
| White | 1616 (54.9) | 402 (54.5) | 401 (55.7) | 427 (57.5) | 386 (51.7) |  |
| Black/Africa American | 316 (10.7) | 103 (14) | 77 (10.7) | 48 (6.5) | 88 (11.8) |  |
| Other | 1014 (34.4) | 232 (31.5) | 242 (33.6) | 267 (36) | 273 (36.5) |  |
| **Vital signs** |  |  |  |  |  |  |
| Heart rate, beats/min | 80.81 ± 15.60 | 79.69 ± 15.09 | 78.74 ± 15.38 | 80.68 ± 15.60 | 84.04 ± 15.81 | < 0.001 |
| Respiratory rate, breaths/min | 19.09 ± 3.40 | 18.68 ± 3.04 | 18.74 ± 3.27 | 19.10 ± 3.38 | 19.80 ± 3.74 | < 0.001 |
| Temperature, °C | 36.88 ± 0.36 | 36.86 ± 0.33 | 36.89 ± 0.30 | 36.90 ± 0.32 | 36.86 ± 0.46 | 0.058 |
| Systolic blood pressure, mmHg | 126.38 ± 18.31 | 128.00 ± 18.39 | 128.64 ± 17.73 | 126.76 ± 18.52 | 122.22 ± 17.91 | < 0.001 |
| Diastolic blood pressure, mmHg | 70.77 ± 12.58 | 71.73 ± 12.53 | 72.55 ± 12.66 | 70.89 ± 12.25 | 67.98 ± 12.45 | < 0.001 |
| SpO₂, % | 96.53 ± 1.84 | 96.55 ± 1.81 | 96.46 ± 1.82 | 96.51 ± 1.85 | 96.59 ± 1.88 | 0.581 |
| **Scoring systems** |  |  |  |  |  |  |
| GCS | 12.87 ± 3.12 | 13.21 ± 2.83 | 13.03 ± 2.89 | 12.84 ± 3.04 | 12.42 ± 3.59 | < 0.001 |
| APSⅢ | 39.98 ± 19.94 | 36.80 ± 17.17 | 35.34 ± 16.98 | 38.55 ± 18.47 | 49.00 ± 23.42 | < 0.001 |
| SAPSⅡ | 30.50 ± 11.68 | 28.56 ± 10.75 | 28.60 ± 11.38 | 30.40 ± 10.61 | 34.36 ± 12.87 | < 0.001 |
| SOFA | 3.0 (1.0, 5.0) | 2.0 (1.0, 4.0) | 2.0 (1.0, 4.0) | 2.0 (1.0, 5.0) | 4.0 (2.0, 7.0) | < 0.001 |
| **Comorbidities** |  |  |  |  |  |  |
| Hypertension | 520 (17.7) | 125 (17) | 122 (16.9) | 133 (17.9) | 140 (18.7) | 0.768 |
| Diabetes | 1208 (41.0) | 385 (52.2) | 222 (30.8) | 241 (32.5) | 360 (48.2) | < 0.001 |
| Myocardial infarct | 806 (27.4) | 155 (21) | 169 (23.5) | 208 (28) | 274 (36.7) | < 0.001 |
| Congestive heart failure | 824 (28.0) | 182 (24.7) | 149 (20.7) | 196 (26.4) | 297 (39.8) | < 0.001 |
| Chronic pulmonary disease | 512 (17.4) | 130 (17.6) | 107 (14.9) | 124 (16.7) | 151 (20.2) | 0.054 |
| Cerebrovascular disease | 1501 (51.0) | 401 (54.4) | 434 (60.3) | 401 (54) | 265 (35.5) | < 0.001 |
| Sepsis | 932 (31.6) | 190 (25.8) | 169 (23.5) | 229 (30.9) | 344 (46.1) | < 0.001 |
| Renal disease | 498 (16.9) | 115 (15.6) | 93 (12.9) | 117 (15.8) | 173 (23.2) | < 0.001 |
| Liver disease | 222 ( 7.5) | 37 (5) | 34 (4.7) | 60 (8.1) | 91 (12.2) | < 0.001 |
| Malignant cancer | 168 ( 5.7) | 42 (5.7) | 22 (3.1) | 44 (5.9) | 60 (8) | < 0.001 |
| **Laboratory tests** |  |  |  |  |  |  |
| WBC (10⁹/L) | 9.65 ± 4.24 | 8.56 ± 3.49 | 8.94 ± 3.56 | 9.99 ± 4.57 | 11.08 ± 4.73 | < 0.001 |
| Hemoglobin (g/dL) | 11.69 ± 2.29 | 11.80 ± 2.21 | 12.04 ± 2.17 | 11.77 ± 2.22 | 11.15 ± 2.44 | < 0.001 |
| Platelet (10⁹/L) | 207.26 ± 77.87 | 213.20 ± 82.13 | 209.16 ± 71.93 | 206.48 ± 72.82 | 200.35 ± 83.34 | 0.014 |
| Sodium (mmol/L) | 136.95 ± 4.68 | 137.23 ± 4.81 | 137.75 ± 4.04 | 137.01 ± 4.45 | 135.83 ± 5.12 | < 0.001 |
| Potassium (mmol/L) | 3.86 ± 0.49 | 3.87 ± 0.50 | 3.85 ± 0.45 | 3.87 ± 0.48 | 3.85 ± 0.54 | 0.824 |
| Calciu(mg/dL) | 8.52 ± 0.76 | 8.57 ± 0.77 | 8.67 ± 0.62 | 8.55 ± 0.74 | 8.29 ± 0.85 | < 0.001 |
| Chloride (mmol/L) | 101.17 ± 5.49 | 101.55 ± 5.56 | 102.00 ± 4.51 | 101.41 ± 5.14 | 99.76 ± 6.30 | < 0.001 |
| BUN (mg/dL) | 15.0 (11.0, 22.0) | 14.0 (10.0, 20.0) | 14.0 (11.0, 19.0) | 15.0 (12.0, 21.0) | 17.0 (13.0, 27.0) | < 0.001 |
| Creatinine (mg/dL) | 0.9 (0.7, 1.1) | 0.8 (0.7, 1.1) | 0.8 (0.7, 1.0) | 0.8 (0.7, 1.1) | 0.9 (0.7, 1.3) | < 0.001 |
| Glucose ,(mg/dL) | 156.90 ± 96.83 | 117.51 ± 47.75 | 122.76 ± 44.56 | 147.25 ± 55.93 | 238.27 ± 142.71 | < 0.001 |
| NLR | 5.3 (2.9, 8.9) | 3.9 (2.4, 6.5) | 4.6 (2.6, 7.4) | 5.9 (3.5, 9.6) | 7.4 (4.1, 13.2) | < 0.001 |
| PLR | 165.6 (104.6, 286.8) | 160.7 (98.3, 277.1) | 158.1 (102.4, 261.6) | 170.2 (111.2, 283.7) | 174.2 (105.3, 321.5) | 0.038 |
| HbA1c(%) | 6.63 ± 2.10 | 7.52 ± 2.65 | 6.21 ± 1.67 | 6.21 ± 1.73 | 6.55 ± 1.93 | < 0.001 |
| SHR | 1.11 ± 0.46 | 0.72 ± 0.12 | 0.93 ± 0.05 | 1.12 ± 0.07 | 1.67 ± 0.58 | < 0.001 |
| **Treatments and Clinical outcomes** |  |  |  |  |  |  |
| Sedative medications | 1131 (38.4) | 233 (31.6) | 268 (37.2) | 289 (38.9) | 341 (45.6) | < 0.001 |
| Corticosteroids | 80 ( 2.7) | 10 (1.4) | 8 (1.1) | 22 (3) | 40 (5.4) | < 0.001 |
| Delirium, n (%) | 619 (21.0) | 107 (14.5) | 111 (15.4) | 173 (23.3) | 228 (30.5) | < 0.001 |
| ICU length of stay, days | 2.7 (1.7, 4.9) | 2.2 (1.6, 3.9) | 2.6 (1.7, 4.5) | 2.9 (1.8, 5.2) | 3.0 (1.8, 5.9) | < 0.001 |
| Hospital length of stay,days | 6.4 (3.7, 11.5) | 5.4 (3.1, 9.7) | 5.6 (3.2, 9.6) | 6.7 (4.0, 11.8) | 8.0 (4.7, 14.2) | < 0.001 |
| ICU mortality | 129 ( 4.4) | 22 (3) | 21 (2.9) | 25 (3.4) | 61 (8.2) | < 0.001 |
| Hospital mortality, n (%) | 192 ( 6.5) | 30 (4.1) | 37 (5.1) | 41 (5.5) | 84 (11.2) | < 0.001 |

Abbreviations: GCS, Glasgow Coma Scale; APS III, Acute Physiology Score III; SAPS II, Simplified Acute Physiology Score II; SOFA, Sequential Organ Failure Assessment; WBC, White Blood Cell count; BUN, Blood Urea Nitrogen; NLR, Neutrophil to Lymphocyte Ratio; PLR, Platelet to Lymphocyte Ratio; HbA1c, Glycated Hemoglobin; SHR, Stress Hyperglycemia Ratio.

Supplementary Table 5 Multivariable logistical regression for SHR and delirium in the nonimputed dataset

| Variable | Model1 | | Model2 | | Model3 | | Model4 | |
| --- | --- | --- | --- | --- | --- | --- | --- | --- |
|  | OR（95％CI） | P | OR（95％CI） | P | OR（95％CI） | P | OR（95％CI） | P |
| SHR | 1.9 (1.58~2.27) | <0.001 | 1.92 (1.61~2.3) | <0.001 | 1.39 (1.09~1.77) | 0.007 | 1.3 (1~1.68) | 0.05 |
| Q1(≤0.85) | 1(Ref) |  | 1(Ref) |  | 1(Ref) |  | 1(Ref) |  |
| Q2(0.86-1.01) | 1.07 (0.8~1.43) | 0.631 | 1.06 (0.79~1.42) | 0.689 | 1.33 (0.86~2.04) | 0.198 | 1.28 (0.8~2.05) | 0.301 |
| Q3(1.02-1.24) | 1.79 (1.37~2.34) | <0.001 | 1.75 (1.33~2.28) | <0.001 | 2.02 (1.36~3) | 0.001 | 2.16 (1.4~3.33) | <0.001 |
| Q4 (≥1.25) | 2.59 (2~3.35) | <0.001 | 2.55 (1.97~3.31) | <0.001 | 2 (1.36~2.95) | <0.001 | 1.92 (1.26~2.94) | 0.003 |
| Trend test |  | <0.001 |  | <0.001 |  | <0.001 |  | 0.001 |

Model1: unadjusted.

Model2: adjusted for age, sex, and race.

Model3: adjusted for model2 + heart rate, respiratory rate, temperature, systolic blood pressure, diastolic blood pressure, oxygen saturation, white blood cell count, hemoglobin, platelet count, sodium, calcium, blood urea nitrogen, creatinine, hypertension, diabetes, congestive heart failure, chronic pulmonary disease, cerebrovascular disease, sepsis, renal disease, liver disease, malignant cancer, platelet-to-lymphocyte ratio, and neutrophil-to-lymphocyte ratio

Model4: adjusted for model3+ Acute Physiology Score III, Simplified Acute Physiology Score II, Glasgow Coma Scale, corticosteroid and sedative use.

Supplementary Table 6 Multivariable logistical regression for SHR and delirium excluded participants with diabetes

| Variable | n(total） | n（ ％ ） | Model1 | | Model2 | | Model3 | | Model4 | |
| --- | --- | --- | --- | --- | --- | --- | --- | --- | --- | --- |
|  |  |  | OR（95％CI） | P | OR（95％CI） | P | OR（95％CI） | P | OR（95％CI） | P |
| SHR | 1738 | 357 (20.5) | 2.62 (1.92~3.59) | <0.001 | 2.75 (2.01~3.77) | <0.001 | 1.68 (1.19~2.36) | 0.003 | 1.64 (1.13~2.39) | 0.01 |
| Q1(≤0.85) | 352 | 50 (14.2) | 1(Ref) |  | 1(Ref) |  | 1(Ref) |  | 1(Ref) |  |
| Q2(0.86-1.01) | 498 | 77 (15.5) | 1.1 (0.75~1.62) | 0.613 | 1.14 (0.77~1.69) | 0.501 | 1.22 (0.79~1.86) | 0.368 | 1.11 (0.7~1.76) | 0.669 |
| Q3(1.02-1.24) | 501 | 106 (21.2) | 1.62 (1.12~2.34) | 0.01 | 1.72 (1.19~2.5) | 0.004 | 1.55 (1.02~2.36) | 0.039 | 1.51 (0.96~2.37) | 0.074 |
| Q4 (≥1.25) | 387 | 124 (32) | 2.85 (1.97~4.11) | <0.001 | 2.99 (2.06~4.34) | <0.001 | 2.03 (1.32~3.13) | 0.001 | 1.92 (1.2~3.07) | 0.007 |
| Trend test | 1738 |  |  | <0.001 |  | <0.001 |  | <0.001 |  | 0.002 |

Model1: unadjusted.

Model2: adjusted for age, sex, and race.

Model3: adjusted for model2 + heart rate, respiratory rate, temperature, systolic blood pressure, diastolic blood pressure, oxygen saturation, white blood cell count, hemoglobin, platelet count, sodium, calcium, blood urea nitrogen, creatinine, hypertension, diabetes, congestive heart failure, chronic pulmonary disease, cerebrovascular disease, sepsis, renal disease, liver disease, malignant cancer, platelet-to-lymphocyte ratio, and neutrophil-to-lymphocyte ratio

Model4: adjusted for model3+ Acute Physiology Score III, Simplified Acute Physiology Score II, Glasgow Coma Scale, corticosteroid and sedative use.

Supplementary Table 7 Multivariable logistical regression for SHR and delirium excluded participants with ICU length of stay > 30 days

| Variable | n(total） | n（ ％ ） | Model1 | | Model2 | | Model3 | | Model4 | |
| --- | --- | --- | --- | --- | --- | --- | --- | --- | --- | --- |
|  |  |  | OR（95％CI） | P | OR（95％CI） | P | OR（95％CI） | P | OR（95％CI） | P |
| SHR | 2924 | 598 (20.5) | 1.9 (1.59~2.28) | <0.001 | 1.93 (1.61~2.32) | <0.001 | 1.4 (1.14~1.73) | 0.002 | 1.24 (0.99~1.55) | 0.064 |
| Q1(≤0.85) | 734 | 105 (14.3) | 1(Ref) |  | 1(Ref) |  | 1(Ref) |  | 1(Ref) |  |
| Q2(0.86-1.01) | 716 | 107 (14.9) | 1.05 (0.79~1.41) | 0.731 | 1.04 (0.78~1.4) | 0.783 | 1.07 (0.77~1.49) | 0.676 | 1 (0.71~1.42) | 0.994 |
| Q3(1.02-1.24) | 733 | 164 (22.4) | 1.73 (1.32~2.26) | <0.001 | 1.68 (1.28~2.21) | <0.001 | 1.53 (1.13~2.08) | 0.006 | 1.54 (1.11~2.13) | 0.01 |
| Q4 (≥1.25) | 741 | 222 (30) | 2.56 (1.98~3.32) | <0.001 | 2.53 (1.94~3.28) | <0.001 | 1.76 (1.3~2.38) | <0.001 | 1.56 (1.12~2.16) | 0.008 |
| Trend test | 2924 |  |  | <0.001 |  | <0.001 |  | <0.001 |  | 0.002 |

Model1: unadjusted.

Model2: adjusted for age, sex, and race.

Model3: adjusted for model2 + heart rate, respiratory rate, temperature, systolic blood pressure, diastolic blood pressure, oxygen saturation, white blood cell count, hemoglobin, platelet count, sodium, calcium, blood urea nitrogen, creatinine, hypertension, diabetes, congestive heart failure, chronic pulmonary disease, cerebrovascular disease, sepsis, renal disease, liver disease, malignant cancer, platelet-to-lymphocyte ratio, and neutrophil-to-lymphocyte ratio

Model4: adjusted for model3+ Acute Physiology Score III, Simplified Acute Physiology Score II, Glasgow Coma Scale, corticosteroid and sedative use.

Supplementary Table 8 Multivariable logistical regression for SHR and delirium excluded participants with SHR > 1.192

| Variable | n(total） | n（ ％ ） | Model1 | | Model2 | | Model3 | | Model4 | |
| --- | --- | --- | --- | --- | --- | --- | --- | --- | --- | --- |
|  |  |  | OR（95％CI） | P | OR（95％CI） | P | OR（95％CI） | P | OR（95％CI） | P |
| SHR | 2076 | 363 (17.5) | 3.68 (1.85~7.3) | <0.001 | 3.27 (1.62~6.57) | 0.001 | 3.17 (1.48~6.77) | 0.003 | 3.21 (1.4~7.34) | 0.006 |

Model1: unadjusted.

Model2: adjusted for age, sex, and race.

Model3: adjusted for model2 + heart rate, respiratory rate, temperature, systolic blood pressure, diastolic blood pressure, oxygen saturation, white blood cell count, hemoglobin, platelet count, sodium, calcium, blood urea nitrogen, creatinine, hypertension, diabetes, congestive heart failure, chronic pulmonary disease, cerebrovascular disease, sepsis, renal disease, liver disease, malignant cancer, platelet-to-lymphocyte ratio, and neutrophil-to-lymphocyte ratio

Model4: adjusted for model3+ Acute Physiology Score III, Simplified Acute Physiology Score II, Glasgow Coma Scale, corticosteroid and sedative use.
